# Introduction and Effectiveness of Early Clinical Exposure Among Dental Students in Pre-clinical Prosthodontics

**DOI:** 10.7759/cureus.66126

**Published:** 2024-08-04

**Authors:** Komal Maheshwari, Kamal Shigli, Bhawana Tiwari, Dwarakananda Bukya, Sukhvinder Singh Oberoi, Mohammad Waseem Faraz Ansari, Tanushree Mondal

**Affiliations:** 1 Department of Prosthodontics, Employees State Insurance Corporation (ESIC) Dental College and Hospital, New Delhi, IND; 2 Department of Prosthodontics, D. Y. Patil Dental School, Pune, IND; 3 Department of Prosthodontics, Employees State Insurance Corporation (ESIC) Dental College and Hospital, Gulbarga, IND; 4 Department of Dentistry, Boston University, Boston, USA; 5 Department of Community Medicine, Employees State Insurance Corporation (ESIC) Dental College and Hospital, Chennai, IND; 6 Department of Community Medicine, Radha Gobinda Kar Medical College and Hospital, Kolkata, IND

**Keywords:** curriculum, pre-clinical prosthodontics, mixed-method, early clinical exposure, dentistry

## Abstract

Background: Lack of patient contact in the pre-clinical prosthodontics curriculum makes it difficult for the students to comprehend and correlate the series of complex steps involved in complete denture fabrication. Early clinical exposure in the second year of the undergraduate program will facilitate a smooth transition of dental students from pre-clinics to clinics, thereby helping to mitigate the lacunae existing in the present curriculum.

Materials and methods: A non-randomized prospective educational interventional study was conducted among 50 second-year dental students. Early clinical exposure in the form of clinical demonstration for complete denture steps of border molding and jaw relation was given subsequent to the completion of didactic and laboratory sessions. Pre- and post-multiple-choice question tests were conducted to explore students’ knowledge. Student perception towards early clinical exposure was assessed using a self-administered anonymous questionnaire, while faculty perception was gauged with the help of in-depth interviews.

Results: A statistically significant difference was observed when pre- and post-multiple-choice question test mean scores were compared, showing an overall improvement in students’ knowledge following early clinical exposure. Students and faculty held a positive perception towards the program and found it to be useful in enhancing the overall learning experience of the students. Faculty expressed their concern regarding limited manpower, paucity of time, and difficulty in integrating early clinical exposure into the present timetable.

Conclusion: Early clinical exposure can be integrated into the traditional pre-clinical prosthodontics curriculum with appropriate time and manpower allocation. Faculty sensitization and training workshops need to be conducted before implementing this program.

## Introduction

The objective of the pre-clinical prosthodontics (PCP) curriculum introduced in the initial two years of the undergraduate dental program is to facilitate a smooth transition of students from the pre-clinical to the clinical phase [1-4]. However, the curriculum falls short of adequately preparing the students due to a lack of patient contact [5]. Students often get perplexed by the complex clinical and laboratory steps involved in the complete denture (CD) fabrication process. This paves the scope for difficulty in visualizing and understanding clinical procedures they have never observed, applying basic theoretical knowledge in clinical settings, and communicating confidently with patients. 

Early clinical exposure (ECE), a teaching-learning methodology that promotes exposure of students to patients as early as in the first year, can help to mitigate the above problem [6]. Studies have found that dental students have expressed a need for ECE [7] and that an amalgamation of the traditional curriculum with ECE facilitated a better understanding of the CD fabrication process, enhanced their educational experience and performance, boosted confidence and improved communication skills [8,9]. In the Indian scenario, we could not identify much literature that has investigated the impact of ECE on the understanding and performance of second-year dental students in PCP, except for published research and an abstract [10,11].

Evidence from these studies facilitated us to conduct a needs assessment survey involving third-year undergraduate students to identify the difficulties faced by them in applying and integrating PCP in clinical settings and to understand their perception regarding the need for the introduction of ECE in the subject of PCP at our institute. The results of the survey highlighted that more than 73% of the students were strongly in favor of the introduction of ECE.

Therefore, the present study was undertaken to assess the effectiveness of the introduction of ECE on the knowledge and understanding of second-year dental students in the subject of PCP.

## Materials and methods

A non-randomized prospective educational interventional study was conducted in the Department of Prosthodontics among second-year Bachelor of Dental Surgery students after obtaining due approval from the Institutional Ethics Committee of the institute. The study was conducted over a period of four months, from July 2022 to October 2022.

Students who attended all the didactic and laboratory sessions related to specific steps in CD fabrication and gave written informed consent were included in the study. Students were recruited using a purposive sampling technique. Of the total number of second-year students (n=58), eight students were selected using computer-generated random numbers for inclusion in the pilot study. The remaining 50 students were included in the main study by complete enumeration.

Class I completely edentulous patients [12] (n=5), aged 65 years or less, both male and female, who were ready for multiple scheduled visits and could establish communication, were selected for inclusion in the study. The detailed study procedure was explained, and patients were recruited once written informed consent was obtained. By the end of the study, all the patients received CDs.

Intervention

Regular CD teaching schedule was followed for all the students. Additionally, students were given ECE for the CD steps of border molding and final impression (FI) and jaw relation (JR) based on the results of the needs assessment survey.

The principal investigator (PI) sensitized the prosthodontic faculty members (n=2) and students toward the concept of ECE. The purpose of the study and the benefits of introducing ECE were discussed. The faculty worked in consensus towards the development and conduct of the program and met prior to the ECE sessions for calibration of their presentations so as to ensure uniformity.

A pilot study was conducted among eight second-year students. They were given clinical demonstrations on a CD patient, and the survey questionnaire was pilot-tested and modified as needed. Data collected from these eight students was not included in the main study.

Study procedure

The study was conducted during pre-clinical theory and practical classes. A multiple-choice question (MCQ) based pre-test was administered to all the students (n=50) after completion of the didactic and laboratory sessions and prior to ECE to ascertain their baseline knowledge. Subsequently, the students were divided into five groups of ten each, and ECE sessions were undertaken by the faculty for each group on a CD patient. An MCQ-based post-test comprising of same questions was administered one week after the completion of ECE sessions. Students’ perceptions towards ECE were assessed using an anonymous, self-administered, pilot-tested questionnaire, and faculty perceptions were recorded using in-depth interviews.

Data collection

MCQ Test

Twenty MCQs were framed for each of the two steps (FI and JR), making a total of 40 MCQs (80 marks: two marks each). Serial numbers of the students were recorded using random sampling for the purpose of anonymity. The MCQs explored the knowledge of the students [13] and were framed to assess the “Knows” (30 out of 40 MCQs) and “Knows How”- clinical context-based (10 out of 40 MCQs) levels of Miller’s pyramid [14]. MCQs were validated by experts in the field, and item analysis was done by administering them to third-year students.

Survey Questionnaire

The anonymous self-administered survey questionnaire comprised two sections: Part A addressed the socio-demographic profile of the students and Part B consisted of 14 items rated on a five-point Likert scale (1=Strongly Disagree; 2=Disagree; 3=Neutral; 4=Agree; 5=Strongly Agree). Items of the questionnaire were developed based on a literature review and discussion among the team members [15,16]. Content validity was confirmed by three clinical professors having experience of more than 14 years. The questionnaire had been pilot-tested to check that all the items were comprehensible and unambiguous. The reliability coefficient, according to Cronbach’s alpha, was 0.882, showing that the overall internal consistency of the questionnaire was good.

In-Depth Interviews

Prosthodontic faculty (n=2) involved in ECE sessions were invited for in-depth face-to-face interviews to explore and gather information regarding their opinions and experiences of the entire process. After obtaining their consent, the PI used a validated interview guide to conduct the interviews and took notes during the discussion. Each interview was audio-recorded to facilitate transcription. The duration of the interviews was 20-30 minutes.

Data analysis

Quantitative data was entered into Microsoft Excel (Microsoft Corporation, Redmond, Washington, USA) and analyzed using Statistical Package for Social Sciences (SPSS) package 26.0 (IBM Corp., Armonk, New York, USA) for relevant statistical comparisons. Results have been presented in the form of tables.

Descriptive statistics was performed by calculating the mean and standard deviation for continuous variables. Categorical variables have been summarized as frequencies and percentages. The satisfaction index was calculated for each item in the questionnaire using the formula given by Bhandari et al. [17]

((n_1_ * 1) + (n_2_ * 2) + (n_4_ * 4) + (n_5_ * 5)) * 20/(n_1_+ n_2_ + n_4_ + n_5_),

where n is the total number of students attaining the score mentioned in the subscript for that individual item.

A paired t-test was used for the comparison of mean scores between two-time intervals (pre- and post-test). The level of statistical significance was set at p-value ≤ 0.05.

Qualitative data was analyzed thematically. Recorded interviews were transcribed using the software Otter.ai and transcripts were prepared. The PI (KM) initiated data analysis by listening to the audio recordings and thoroughly reading the transcripts. Broad codes were identified, and further analysis and linking of the coded data was done to develop themes. Finally, the main themes that emerged during the analysis were articulated. The researcher (KS) was blinded to the themes and made to go through the transcripts to check with the codes and themes arrived at by KM. Any differences in interpretation were resolved through discussion. Their pre-understanding and experiences were bracketed to avoid personal bias. Credibility was established by member checks, and for the transferability of findings, segments of verbatim quotes from the participants have been included, which also provides contextual material to support the emergent themes. 

## Results

Quantitative data

All the students (n=50) participated in the study, took the pre-post MCQ tests and returned duly filled survey forms. The mean age of the respondents was 21.38±0.90 years and 66% of the respondents were females.

Table 1 shows a comparison of the pre- and post-MCQ test mean scores. The overall mean score changed from 42.96±11.00 in the pre-test to 54.08±10.09 in the post-test. The mean scores obtained for FI changed from 22.88±6.85 in the pre-test to 28.52±6.00 in the post-test and that for JR from 20.08±5.48 in the pre-test to 25.56±5.44 in the post-test. Overall, FI and JR mean scores increased significantly from the pre-test to the post-test (p-value <0.05).

**Table 1:** Comparison of the pre- and post-MCQ test mean scores. The mean overall, FI, and JR scores were compared between the pre- and post-test using the paired t-test. *The level of statistical significance was set at a p-value less than or equal to 0.05. FI: final impression, JR: jaw relation, MCQ: multiple-choice question.

Scores	MCQ test	Mean score	Std. deviation	p-value* (<0.05)
Overall	Pre-test	42.96	11.00	0.001
	Post-test	54.08	10.09	
FI	Pre-test	22.88	6.85	0.001
	Post-test	28.52	6.00	
JR	Pre-test	20.08	5.48	0.001
	Post-test	25.56	5.44	

Table 2 shows descriptive statistics of students’ perception and satisfaction index towards ECE. For all the items except item 10, the satisfaction index was more than 90 on a scale of 1-100. It was highest (97.6) for item 13 and lowest (87.8) for item 10. The satisfaction index is indicative that students were highly satisfied with the ECE program and preferred that ECE should be included in the PCP curriculum.

**Table 2:** Students’ perception and satisfaction towards ECE. §Satisfaction index calculation: ((n_1_ * 1) + (n_2_ * 2) + (n_4_ * 4) + (n_5_ * 5)) * 20/(n_1_ + n_2_+ n_4_+ n_5_)), Bhandari et al. [17]. ECE: early clinical exposure.

Survey items	Likert scale responses	Frequency	Percentage (%)	Satisfaction index^§^
1. This method of teaching has stimulated interest in the subject	Neutral–3	2	4.0%	93.75
	Agree–4	15	30.0%	
	Strongly agree–5	33	66.0%	
2. ECE has enabled a better understanding of the discussed complete denture procedures	Agree–4	18	36.0%	92.8
	Strongly agree–5	32	64.0%	
3. ECE has enhanced your knowledge of the subject than what it was priorly	Neutral–3	1	2.0%	92.2
	Agree–4	19	38.0%	
	Strongly agree–5	30	60.0%	
4. ECE has allowed better comprehension of the acquired knowledge	Agree–4	23	46.0%	90.8
	Strongly agree–5	27	54.0%	
5. ECE will help you to effectively apply basic theoretical knowledge in a clinical context	Neutral–3	1	2.0%	90.6
	Agree–4	23	46.0%	
	Strongly agree–5	26	52.0%	
6. ECE will help in better retention of the topics as compared to the traditional curriculum	Neutral–3	4	8.0%	91.3
	Agree–4	20	40.0%	
	Strongly agree–5	26	52.0%	
7. ECE will help in better recall of the topics as compared to the traditional curriculum	Neutral–3	5	10.0%	92
	Agree–4	18	36.0%	
	Strongly agree–5	27	54.0%	
8. ECE has motivated you to learn more about complete denture prosthodontics	Neutral–3	11	22.0%	90.2
	Agree–4	19	38.0%	
	Strongly agree – 5	20	40.0%	
9. ECE has raised your confidence prior to clinical exposure in the third year	Agree – 4	25	50.0%	90
	Strongly agree–5	25	50.0%	
10. ECE will help you to establish effective communication with patients in the future	Neutral–3	9	18.0%	87.8
	Agree–4	25	50.0%	
	Strongly agree–5	16	32.0%	
11. There was adequate interaction between the students and the faculty during ECE	Disagree–2	1	2.0%	91.9
	Neutral–3	3	6.0%	
	Agree–4	16	32.0%	
	Strongly agree–5	30	60.0%	
12. ECE was enjoyable and you are satisfied with this method of teaching	Neutral–3	2	4.0%	93.3
	Agree–4	16	32.0%	
	Strongly agree–5	32	64.0%	
13. You will prefer this method of teaching to be included in the pre-clinical prosthodontics curriculum	Agree–4	6	12.0%	97.6
	Strongly agree–5	44	88.0%	
14. You would like to learn other topics in prosthodontics with ECE	Agree–4	9	18.0%	96.4
	Strongly agree–5	41	82.0%	

Qualitative data

Thematic inductive analysis of faculty responses to the in-depth interview was carried out, and results have been presented in Table 3. The main themes that have emerged are faculty satisfaction, beneficial for students and patients, faculty shortage, time-consuming, difficult to integrate into the present timetable, other departmental and academic activities affected, increased faculty-student interaction, and use of video-assisted teaching learning in large groups.

**Table 3:** Main themes from faculty responses to the in-depth interview. ECE: early clinical exposure.

Sr. no	Question	Verbatim responses	Themes
1.	How satisfied are you with the overall teaching experience?	“Very well-planned exercise,” “I was very happy with the way we gave them the clinical exposure; it was a very good experience”	Faculty satisfied with ECE experience
2.	What do you think are the strengths of ECE?	“Student is exposed to the clinical aspect of the border molding and jaw relation, which they usually find very difficult when they get into the third year,” “They also had a chance to in fact, interact with the patient and ask him the problems, what he faced with the edentulism, or why he had come to the clinics”	Beneficial for students, Beneficial for patients
3.	What do you think are the challenges that you faced with ECE?	“Student-faculty ratio is not very healthy,” “Another challenge is that it is very time-consuming also to give demonstration to groups,” “Number of hours dedicated for labs and other classes is limited, so how it will be adjusted in the other years also, apart from this study, it's a big challenge	Faculty shortage, Time consuming, Difficult to integrate into the timetable, Other departmental and academic activities affected
4.	Can you talk about your level of interaction with students during ECE versus traditional curriculum?	“We made a group of 10 students to one demonstration. So, that it helps more, each student can participate and ask questions,” “They also had a chance to get their doubts cleared then and there”	Increased faculty-student interaction, Immediate resolution of doubts
5.	Any other suggestions?	“I think if not like one faculty to one group of students, if it can be extended in some other way like, we can have that video recording which we can play in the clinic, clinical recording whatever cases were presented to a larger group” “We would ask the students to come thoroughly prepared about these topics”	Video-assisted teaching, learning in large groups, Students should go through reading material prior to ECE

## Discussion

The present study aimed to assess the effectiveness of the introduction of ECE on the knowledge and understanding of students in the subject of PCP through clinical demonstration on real patients. It has been stated that learning through real patient contact leads to higher metacognitive awareness among medical students and enhanced learning outcomes [18]. Direct contact with patients can play an important role in the development of communication skills, professional outlook, and empathy towards patients [19].

Students’ knowledge: There was a significant increase in student's knowledge when pre- and post-MCQ test scores were compared in the present study. Similar results have been reported in other studies conducted among dental and medical students [9,10,20].

Students’ perception towards ECE: It was recorded using the survey questionnaire and ECE was perceived positively by the students. The majority of the respondents agreed that ECE stimulated interest in the subject (96%) and enabled a better understanding of the discussed procedures (100%). Enhanced student interest and understanding have been reported when clinical exposure is provided to students in the early years of dental and medical education programs [7,8,15,16,20-25].

Students in our study held a strong perception that ECE enhanced their knowledge of the subject than what it was priorly (98%). In a study by Patil et al., dental students with ECE appeared to have better self-perception regarding subject knowledge as compared to the late clinical exposure students [26].

Better understanding with improved knowledge, comprehension, application as well as its retention is important for student learning. It encourages and motivates students and boosts their confidence. Responses obtained from the students pointed out that ECE allowed; better comprehension (100%) and application of gained knowledge in the clinical context (98%) and better retention (92%) and recall of the topics (90%). Similar results have been reported in studies published in dental [10,23] and medical literature [15,16,20]. Students also reported that ECE motivated them to learn more about CD prosthodontics (78%), which correlates with the findings from studies by Shigli et al. and Adisman [10,21]. Medical students have also reported that ECE motivated them in various ways, including by improving their knowledge, clinical and communication skills, and confidence [15,24,25].

Students also agreed that ECE; facilitated adequate faculty-student interaction (92%), it was enjoyable (96%) and they would like to learn other topics with this method (100%). Similar findings have been reported in both dental [27] and medical literature [15,16].

Students perceived that ECE raised their confidence prior to clinical exposure in the third year (100%). A similar result has been reported by Lang et al., wherein ECE boosted dental students’ confidence to handle their patients’ problems in practice [27]. About 82% of the students in our study agreed with the statement, “ECE will help to establish effective communication with patients in the future." Few studies have also reported that ECE helps to improve students’ communication skills [19,28]. All the students agreed to the statement, “You will prefer this method of teaching to be included in the pre-clinical prosthodontics curriculum." A similar result has been reported in the study by Shigli et al. [10].

Faculty perceptions towards ECE: It were recorded by conducting in-depth interviews. Overall, the faculty was very satisfied with the experience of planning and conducting the ECE program. Faculty also expressed satisfaction with their experience working with cooperative patients and enthusiastic students.

Faculty considered ECE to be beneficial both for the students as well as the patients. ECE facilitated an understanding of the clinical aspects of CD steps and their correlation with the theoretical aspects. Studies show that ECE provides context to theoretical learning and helps students to apply knowledge in future clinical practice [10,23].

Moreover, ECE may also facilitate better clinical performance by the students as it helps them to understand how to conduct a particular clinical procedure. This is in consensus with the results from the study by Ewnte and Yigzaw [24] and Wenrich et al. [29], who reported that ECE is helpful in familiarizing students with patients and the development of clinical skills.

ECE benefits patients by enhancing students' understanding of their problems and needs, as well as through students’ improved clinical acumen. In the present study, patients received CDs as a result of the faculty's direct involvement through clinical demonstration to the students. 

The major challenge perceived by faculty was the low faculty-student ratio, due to which other departmental and academic activities may get affected because of their involvement in ECE. Also, it is a time-consuming method and is difficult to integrate into the present timetable. Similar challenges have been reported in studies by Dehghan et al. [20] and Ewnte and Yigzaw [24].

In the present study, faculty felt that ECE encouraged active student interaction and participation, provided students an opportunity to raise doubts, and facilitated their immediate resolution. ECE is helpful in the development of interpersonal communication skills through interaction with patients and dental team members [23]. The use of video recordings of clinical demonstrations in large group teaching-learning sessions was suggested by the faculty to overcome the challenges of limited manpower and time.

Limitations and future recommendations

It is a single-center study limited to a single batch of second-year dental students; therefore, the results are not generalizable. Intermediate and long-term outcomes pertaining to improvement in students’ skills, retention and recall of knowledge, and patient care and satisfaction have not been explored. A multi-centric study can be planned and conducted involving different dental institutions across India to achieve comprehensive outcomes, which will facilitate further action regarding the inclusion of ECE in the undergraduate dental curriculum in the subject of PCP.

## Conclusions

The introduction of ECE in the subject of PCP at our institute has been instrumental in significantly improving students’ knowledge and understanding of CD clinical procedures. Overall, both the students and faculty were satisfied with the new experience. However, the availability of adequate manpower and allocation of dedicated hours for ECE in the existing timetable needs to be considered before including it in the present curriculum. Also, faculty sensitization and training workshops need to be conducted before implementing this program.
